# 3-Benzamido-1-benzoyl-1*H*-pyrrol-2(5*H*)-one

**DOI:** 10.1107/S1600536810006483

**Published:** 2010-02-24

**Authors:** Marta Kasunič, Bojan Verček, Irena Mušič, Amalija Golobič

**Affiliations:** a Faculty of Chemistry and Chemical Technology, University of Ljubljana, Aškerčeva 5, 1000 Ljubljana, Slovenia

## Abstract

In the title compound, C_18_H_14_N_2_O_3_, one of the phenyl rings is almost coplanar with the pyrrole ring [dihedral angle = 2.56 (14)°], whereas the other one is tilted by 63.01 (6)° with respect to the pyrrole ring. Since the NH group is shielded from possible acceptors, this group is not involved in hydrogen bonding.

## Related literature

For the synthesis of 1,5-dihydro-2*H*-pyrrol-2-ones, see: Gao *et al.* (1997 ▶); Alizadeh *et al.* (2006 ▶); Nedolya *et al.* (2002 ▶); Mušič *et al.* (1998 ▶).
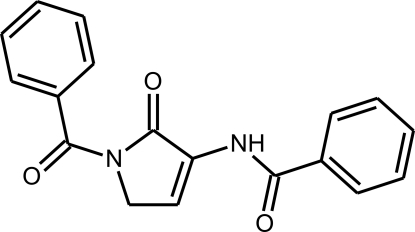


## Experimental

### 

#### Crystal data

C_18_H_14_N_2_O_3_
*M* *_r_* = 306.31
Monoclinic, 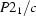

*a* = 20.966 (2) Å
*b* = 5.8891 (7) Å
*c* = 12.329 (1) Å
β = 95.908 (8)°
*V* = 1514.2 (3) Å^3^
*Z* = 4
Mo *K*α radiation
μ = 0.09 mm^−1^
*T* = 293 K
0.58 × 0.36 × 0.09 mm

#### Data collection

Enraf–Nonius CAD-4 diffractometer
13450 measured reflections
3648 independent reflections
2119 reflections with *I* > 2σ(*I*)
*R*_int_ = 0.056
3 standard reflections every 333.3 min  intensity decay: 1.1%

#### Refinement

*R*[*F* ^2^ > 2σ(*F* ^2^)] = 0.046
*wR*(*F* ^2^) = 0.132
*S* = 1.00
3648 reflections
209 parameters
H-atom parameters constrained
Δρ_max_ = 0.16 e Å^−3^
Δρ_min_ = −0.18 e Å^−3^

### 

Data collection: *CAD-4 Software* (Enraf–Nonius, 1989 ▶); cell refinement: the *XRAY76* System (Stewart *et al.*, 1976 ▶); data reduction: *XCAD4* (Harms & Wocadlo, 1995 ▶); program(s) used to solve structure: *SIR92* (Altomare *et al.*, 1993 ▶); program(s) used to refine structure: *SHELXL97* (Sheldrick, 2008 ▶); molecular graphics: *ORTEP-3* (Farrugia, 1997 ▶); software used to prepare material for publication: *SHELXL97*.

## Supplementary Material

Crystal structure: contains datablocks I, global. DOI: 10.1107/S1600536810006483/bt5197sup1.cif

Structure factors: contains datablocks I. DOI: 10.1107/S1600536810006483/bt5197Isup2.hkl

Additional supplementary materials:  crystallographic information; 3D view; checkCIF report

## Figures and Tables

**Fig. 1.**
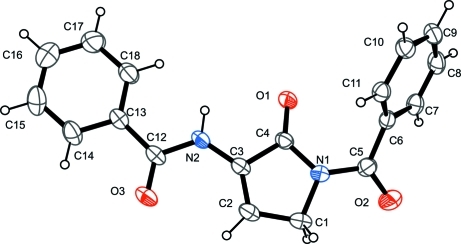
The molecular structure of the title compound, showing the atom-labelling scheme. Displacement ellipsoids are drawn at the 30% probability level and H atoms are drawn as small spheres of arbitrary radii.

## supplementary crystallographic information

### Comment

1,5-dihydro-2H-pyrrol-2-ones comprise a family of lactams which are found as substructures in several natural products with promising pharmalogical properties. Several reports on synthesis of substituted 1,5-dihydro-2H-pyrrol-2-ones exist and these compounds can be prepared *via* different synthetic pathways (e.g. Gao *et al.*, 1997; Alizadeh *et al.*, 2006; Nedolya *et al.*, 2002), Mušič *et al.* (1998). The asymmetry unit of the title compound with atom labelling scheme can be seen in Fig. 1.

### Experimental

The title compound was prepared according to the procedure by Mušič *et al.* (1998). The crystals, suitable for X-ray structure analysis, were obtained by slow crystallization from the mixture of acetonitrile and hexane at room temperature.

### Refinement

All H atoms were positioned geometrically and allowed to ride on their parent atoms [C—H = 0.97 for methylene and 0.93 Å for aromatic hydrogens, respectively, N—H = 0.86 Å and *U*_iso_(H) = 1.2 times *U*_eq_(C, N)].

### Crystal data

**Table:**

C_18_H_14_N_2_O_3_	*F*(000) = 640
*M**_r_* = 306.31	*D*_x_ = 1.344 Mg m^−^^3^
Monoclinic, *P*2_1_/*c*	Mo *K*α radiation, λ = 0.71073 Å
Hall symbol: -P 2ybc	Cell parameters from 75 reflections
*a* = 20.966 (2) Å	θ = 8.0–15.3°
*b* = 5.8891 (7) Å	µ = 0.09 mm^−^^1^
*c* = 12.329 (1) Å	*T* = 293 K
β = 95.908 (8)°	Plate, pale yellow
*V* = 1514.2 (3) Å^3^	0.58 × 0.36 × 0.09 mm
*Z* = 4	

### Data collection

**Table:**

Enraf–Nonius CAD-4 diffractometer	*R*_int_ = 0.056
Radiation source: fine-focus sealed tube	θ_max_ = 28.0°, θ_min_ = 2.0°
graphite	*h* = −27→27
ω/2θ scans [width (0.85 + 0.3tanθ)]	*k* = −7→7
13450 measured reflections	*l* = −16→14
3648 independent reflections	3 standard reflections every 333.3 min
2119 reflections with *I* > 2σ(*I*)	intensity decay: 1.1%

### Refinement

**Table:**

Refinement on *F*^2^	Secondary atom site location: difference Fourier map
Least-squares matrix: full	Hydrogen site location: inferred from neighbouring sites
*R*[*F*^2^ > 2σ(*F*^2^)] = 0.046	H-atom parameters constrained
*wR*(*F*^2^) = 0.132	*w* = 1/[σ^2^(*F*_o_^2^) + (0.0676*P*)^2^ + 0.1617*P*] where *P* = (*F*_o_^2^ + 2*F*_c_^2^)/3
*S* = 1.00	(Δ/σ)_max_ = 0.001
3648 reflections	Δρ_max_ = 0.16 e Å^−^^3^
209 parameters	Δρ_min_ = −0.18 e Å^−^^3^
0 restraints	Extinction correction: *SHELX97* (Sheldrick, 2008), Fc^*^=kFc[1+0.001xFc^2^λ^3^/sin(2θ)]^-1/4^
Primary atom site location: structure-invariant direct methods	Extinction coefficient: 0.029 (3)

### Special details

**Table:**

Geometry. All esds (except the esd in the dihedral angle between two l.s. planes) are estimated using the full covariance matrix. The cell esds are taken into account individually in the estimation of esds in distances, angles and torsion angles; correlations between esds in cell parameters are only used when they are defined by crystal symmetry. An approximate (isotropic) treatment of cell esds is used for estimating esds involving l.s. planes.
Refinement. Refinement of *F*^2^ against ALL reflections. The weighted *R*-factor wR and goodness of fit *S* are based on *F*^2^, conventional *R*-factors *R* are based on *F*, with *F* set to zero for negative *F*^2^. The threshold expression of *F*^2^ > σ(*F*^2^) is used only for calculating *R*-factors(gt) etc. and is not relevant to the choice of reflections for refinement. *R*-factors based on *F*^2^ are statistically about twice as large as those based on *F*, and *R*- factors based on ALL data will be even larger.

### Fractional atomic coordinates and isotropic or equivalent isotropic displacement parameters (Å^2^)

**Table:**

	*x*	*y*	*z*	*U*_iso_*/*U*_eq_	
N1	0.80491 (6)	0.1771 (2)	0.90089 (10)	0.0492 (3)	
N2	0.69375 (7)	0.6347 (2)	0.88815 (11)	0.0554 (4)	
H2	0.6985	0.6820	0.8235	0.066*	
O1	0.77372 (6)	0.4192 (2)	0.75402 (9)	0.0632 (4)	
O2	0.85617 (8)	−0.1579 (2)	0.90309 (12)	0.0846 (5)	
O3	0.64221 (7)	0.6962 (3)	1.03607 (10)	0.0784 (4)	
C1	0.78338 (8)	0.1439 (3)	1.00943 (12)	0.0534 (4)	
H1A	0.8190	0.1548	1.0660	0.064*	
H1B	0.7627	−0.0024	1.0147	0.064*	
C2	0.73736 (8)	0.3319 (3)	1.01751 (13)	0.0550 (4)	
H2A	0.7162	0.3627	1.0785	0.066*	
C3	0.73060 (7)	0.4501 (3)	0.92579 (12)	0.0483 (4)	
C4	0.77164 (7)	0.3562 (3)	0.84693 (12)	0.0472 (4)	
C5	0.84667 (8)	0.0250 (3)	0.85911 (13)	0.0534 (4)	
C6	0.88045 (7)	0.0966 (3)	0.76467 (12)	0.0453 (4)	
C7	0.88460 (8)	−0.0552 (3)	0.67966 (14)	0.0549 (4)	
H7	0.8640	−0.1950	0.6800	0.066*	
C8	0.91946 (9)	0.0018 (3)	0.59465 (14)	0.0634 (5)	
H8	0.9210	−0.0975	0.5363	0.076*	
C9	0.95206 (9)	0.2049 (3)	0.59581 (15)	0.0619 (5)	
H9	0.9763	0.2408	0.5391	0.074*	
C10	0.94882 (8)	0.3549 (3)	0.68073 (14)	0.0555 (4)	
H10	0.9712	0.4913	0.6817	0.067*	
C11	0.91240 (7)	0.3029 (3)	0.76453 (12)	0.0487 (4)	
H11	0.9093	0.4061	0.8208	0.058*	
C12	0.65088 (8)	0.7482 (3)	0.94360 (13)	0.0528 (4)	
C13	0.61696 (8)	0.9416 (3)	0.88463 (13)	0.0512 (4)	
C14	0.57527 (10)	1.0680 (4)	0.93989 (16)	0.0712 (6)	
H14	0.5678	1.0268	1.0103	0.085*	
C15	0.54448 (12)	1.2550 (4)	0.8915 (2)	0.0884 (7)	
H15	0.5166	1.3397	0.9294	0.106*	
C16	0.55488 (11)	1.3153 (4)	0.7884 (2)	0.0860 (7)	
H16	0.5347	1.4425	0.7561	0.103*	
C17	0.59485 (12)	1.1893 (5)	0.7325 (2)	0.0956 (8)	
H17	0.6015	1.2299	0.6616	0.115*	
C18	0.62549 (10)	1.0025 (4)	0.77981 (16)	0.0770 (6)	
H18	0.6523	0.9165	0.7403	0.092*	

### Atomic displacement parameters (Å^2^)

**Table:**

	*U*^11^	*U*^22^	*U*^33^	*U*^12^	*U*^13^	*U*^23^
N1	0.0550 (8)	0.0540 (8)	0.0394 (7)	0.0026 (7)	0.0086 (6)	0.0046 (6)
N2	0.0570 (8)	0.0713 (10)	0.0406 (7)	0.0103 (7)	0.0184 (6)	0.0029 (7)
O1	0.0735 (8)	0.0808 (9)	0.0375 (6)	0.0250 (7)	0.0167 (5)	0.0084 (6)
O2	0.1141 (12)	0.0581 (8)	0.0851 (10)	0.0181 (8)	0.0264 (9)	0.0236 (7)
O3	0.0878 (10)	0.1034 (11)	0.0492 (7)	0.0198 (8)	0.0315 (7)	0.0071 (7)
C1	0.0575 (10)	0.0645 (11)	0.0387 (8)	−0.0111 (8)	0.0071 (7)	0.0064 (8)
C2	0.0519 (9)	0.0741 (11)	0.0409 (8)	−0.0055 (9)	0.0139 (7)	0.0004 (8)
C3	0.0457 (8)	0.0620 (11)	0.0385 (8)	−0.0033 (8)	0.0105 (6)	−0.0017 (7)
C4	0.0489 (9)	0.0573 (10)	0.0361 (8)	0.0018 (8)	0.0075 (6)	−0.0009 (7)
C5	0.0589 (10)	0.0492 (10)	0.0515 (9)	0.0025 (8)	0.0031 (8)	0.0027 (8)
C6	0.0466 (8)	0.0432 (8)	0.0457 (8)	0.0093 (7)	0.0027 (6)	−0.0018 (7)
C7	0.0574 (10)	0.0444 (9)	0.0625 (11)	0.0076 (8)	0.0048 (8)	−0.0092 (8)
C8	0.0682 (12)	0.0659 (12)	0.0566 (11)	0.0189 (10)	0.0093 (9)	−0.0179 (9)
C9	0.0539 (10)	0.0758 (13)	0.0584 (11)	0.0125 (9)	0.0168 (8)	0.0003 (9)
C10	0.0486 (9)	0.0561 (10)	0.0624 (10)	−0.0002 (8)	0.0089 (8)	0.0008 (8)
C11	0.0514 (9)	0.0466 (9)	0.0475 (9)	0.0047 (7)	0.0026 (7)	−0.0060 (7)
C12	0.0484 (9)	0.0688 (11)	0.0432 (9)	−0.0040 (8)	0.0152 (7)	−0.0079 (8)
C13	0.0446 (8)	0.0621 (10)	0.0479 (9)	−0.0041 (8)	0.0098 (7)	−0.0112 (8)
C14	0.0744 (13)	0.0822 (14)	0.0590 (11)	0.0110 (11)	0.0160 (9)	−0.0205 (10)
C15	0.0897 (17)	0.0788 (15)	0.0968 (17)	0.0231 (13)	0.0096 (13)	−0.0297 (13)
C16	0.0808 (16)	0.0710 (14)	0.1039 (19)	0.0063 (12)	−0.0011 (13)	0.0066 (13)
C17	0.0862 (16)	0.116 (2)	0.0873 (16)	0.0289 (15)	0.0238 (13)	0.0341 (15)
C18	0.0704 (12)	0.1031 (16)	0.0608 (11)	0.0271 (12)	0.0233 (10)	0.0130 (11)

### Geometric parameters (Å, °)

**Table:**

N1—C5	1.388 (2)	C8—C9	1.377 (3)
N1—C4	1.395 (2)	C8—H8	0.9300
N1—C1	1.4687 (19)	C9—C10	1.377 (2)
N2—C12	1.360 (2)	C9—H9	0.9300
N2—C3	1.385 (2)	C10—C11	1.381 (2)
N2—H2	0.8600	C10—H10	0.9300
O1—C4	1.2091 (18)	C11—H11	0.9300
O2—C5	1.213 (2)	C12—C13	1.492 (2)
O3—C12	1.2121 (19)	C13—C18	1.370 (2)
C1—C2	1.479 (3)	C13—C14	1.380 (2)
C1—H1A	0.9700	C14—C15	1.381 (3)
C1—H1B	0.9700	C14—H14	0.9300
C2—C3	1.323 (2)	C15—C16	1.358 (3)
C2—H2A	0.9300	C15—H15	0.9300
C3—C4	1.471 (2)	C16—C17	1.359 (3)
C5—C6	1.485 (2)	C16—H16	0.9300
C6—C11	1.387 (2)	C17—C18	1.373 (3)
C6—C7	1.387 (2)	C17—H17	0.9300
C7—C8	1.379 (2)	C18—H18	0.9300
C7—H7	0.9300		
			
C5—N1—C4	127.91 (13)	C7—C8—H8	119.8
C5—N1—C1	121.13 (13)	C8—C9—C10	120.13 (17)
C4—N1—C1	110.46 (13)	C8—C9—H9	119.9
C12—N2—C3	126.31 (14)	C10—C9—H9	119.9
C12—N2—H2	116.8	C9—C10—C11	120.07 (17)
C3—N2—H2	116.8	C9—C10—H10	120.0
N1—C1—C2	103.04 (13)	C11—C10—H10	120.0
N1—C1—H1A	111.2	C10—C11—C6	119.96 (15)
C2—C1—H1A	111.2	C10—C11—H11	120.0
N1—C1—H1B	111.2	C6—C11—H11	120.0
C2—C1—H1B	111.2	O3—C12—N2	121.29 (17)
H1A—C1—H1B	109.1	O3—C12—C13	122.77 (15)
C3—C2—C1	110.47 (14)	N2—C12—C13	115.92 (14)
C3—C2—H2A	124.8	C18—C13—C14	118.38 (18)
C1—C2—H2A	124.8	C18—C13—C12	123.87 (16)
C2—C3—N2	134.92 (15)	C14—C13—C12	117.74 (16)
C2—C3—C4	110.40 (15)	C15—C14—C13	120.6 (2)
N2—C3—C4	114.67 (13)	C15—C14—H14	119.7
O1—C4—N1	128.21 (15)	C13—C14—H14	119.7
O1—C4—C3	126.27 (15)	C16—C15—C14	120.0 (2)
N1—C4—C3	105.48 (13)	C16—C15—H15	120.0
O2—C5—N1	119.19 (16)	C14—C15—H15	120.0
O2—C5—C6	122.21 (16)	C15—C16—C17	119.9 (2)
N1—C5—C6	118.54 (14)	C15—C16—H16	120.0
C11—C6—C7	119.70 (15)	C17—C16—H16	120.0
C11—C6—C5	121.26 (14)	C16—C17—C18	120.5 (2)
C7—C6—C5	118.79 (15)	C16—C17—H17	119.7
C8—C7—C6	119.78 (17)	C18—C17—H17	119.7
C8—C7—H7	120.1	C13—C18—C17	120.6 (2)
C6—C7—H7	120.1	C13—C18—H18	119.7
C9—C8—C7	120.31 (16)	C17—C18—H18	119.7
C9—C8—H8	119.8		
			
C5—N1—C1—C2	−176.46 (15)	C11—C6—C7—C8	−1.2 (2)
C4—N1—C1—C2	−3.92 (17)	C5—C6—C7—C8	−175.60 (15)
N1—C1—C2—C3	2.68 (19)	C6—C7—C8—C9	2.4 (3)
C1—C2—C3—N2	178.14 (18)	C7—C8—C9—C10	−1.5 (3)
C1—C2—C3—C4	−0.6 (2)	C8—C9—C10—C11	−0.6 (3)
C12—N2—C3—C2	−0.5 (3)	C9—C10—C11—C6	1.8 (2)
C12—N2—C3—C4	178.20 (15)	C7—C6—C11—C10	−0.9 (2)
C5—N1—C4—O1	−2.2 (3)	C5—C6—C11—C10	173.38 (15)
C1—N1—C4—O1	−174.11 (17)	C3—N2—C12—O3	1.6 (3)
C5—N1—C4—C3	175.56 (15)	C3—N2—C12—C13	−179.80 (15)
C1—N1—C4—C3	3.67 (17)	O3—C12—C13—C18	−179.38 (19)
C2—C3—C4—O1	175.90 (17)	N2—C12—C13—C18	2.0 (3)
N2—C3—C4—O1	−3.1 (3)	O3—C12—C13—C14	1.6 (3)
C2—C3—C4—N1	−1.93 (19)	N2—C12—C13—C14	−177.04 (16)
N2—C3—C4—N1	179.08 (13)	C18—C13—C14—C15	−1.9 (3)
C4—N1—C5—O2	−157.39 (17)	C12—C13—C14—C15	177.23 (18)
C1—N1—C5—O2	13.7 (2)	C13—C14—C15—C16	0.4 (3)
C4—N1—C5—C6	25.3 (2)	C14—C15—C16—C17	1.0 (4)
C1—N1—C5—C6	−163.60 (14)	C15—C16—C17—C18	−0.7 (4)
O2—C5—C6—C11	−127.91 (19)	C14—C13—C18—C17	2.1 (3)
N1—C5—C6—C11	49.3 (2)	C12—C13—C18—C17	−176.9 (2)
O2—C5—C6—C7	46.4 (2)	C16—C17—C18—C13	−0.9 (4)
N1—C5—C6—C7	−136.36 (16)		
